# Hypertension and Related Comorbidities as Potential Risk Factors for COVID-19 Hospitalization and Severity: A Prospective Population-Based Cohort Study

**DOI:** 10.3390/jcm10061194

**Published:** 2021-03-12

**Authors:** Ujué Fresán, Marcela Guevara, Camino Trobajo-Sanmartín, Cristina Burgui, Carmen Ezpeleta, Jesús Castilla

**Affiliations:** 1 Instituto de Salud Pública de Navarra, 31003 Pamplona, Spain; mp.guevara.eslava@navarra.es (M.G.); cristina.burgui.alcaide@navarra.es (C.B.); jcastilc@navarra.es (J.C.); 2 CIBER Epidemiología y Salud Pública (CIBERESP), 28029 Madrid, Spain; 3 Instituto de Investigación Sanitaria de Navarra (IdiSNA), 31008 Pamplona, Spain; camino.trobajo.sanmartin@navarra.es (C.T.-S.); carmen.ezpeleta.baquedano@navarra.es (C.E.); 4 e-Health Group, Instituto de Salud Global Barcelona (ISGlobal), 08003 Barcelona, Spain; 5 Servicio de Microbiología Clínica, Complejo Hospitalario de Navarra, 31008 Pamplona, Spain

**Keywords:** hypertension, cardiovascular disease, cerebrovascular disease, chronic kidney disease, SARS-CoV-2, COVID-19 hospitalization, COVID-19 mortality

## Abstract

The independent role of hypertension for COVID-19 outcomes in the population remains unclear. We aimed to estimate the independent effect of hypertension and hypertension-related conditions, i.e., cardiovascular, cerebrovascular and chronic kidney diseases, as potential risk factors for COVID-19 hospitalization and severe COVID-19 (i.e., intensive care unit admission or death) in the population. The risk for severe COVID-19 among hospitalized patients was also evaluated. A Spanish population-based cohort of people aged 25–79 years was prospectively followed from March to May 2020 to identify hospitalizations for laboratory-confirmed COVID-19. Poisson regression was used to estimate the adjusted relative risk (aRR) for COVID-19 hospitalization and severe COVID-19 among the whole cohort, and for severe COVID-19 among hospitalized patients. Of 424,784 people followed, 1106 were hospitalized by COVID-19 and 176 were severe cases. Hypertension was not independently associated with a higher risk of hospitalization (aRR 0.96, 95% CI 0.83–1.12) nor severe COVID-19 (aRR 1.12, 95% CI 0.80–1.56) in the population. Persons with cardiovascular, cerebrovascular and chronic kidney diseases were at higher risk for COVID-19 hospitalization (aRR 1.33, 95% CI 1.13–1.58; aRR 1.41, 95% CI 1.04–1.92; and aRR 1.52, 95% CI 1.21–1.91; respectively) and severe COVID-19 (aRR 1.61, 95% CI 1.13–2.30; aRR 1.91, 95% CI 1.13–3.25; and aRR 1.78, 95% CI 1.14–2.76; respectively). COVID-19 hospitalized patients with cerebrovascular diseases were at higher risk of mortality (aRR 1.80, 95% CI 1.00–3.23). The current study shows that, in the general population, persons with cardiovascular, cerebrovascular and chronic kidney diseases, but not those with hypertension only, should be considered as high-risk groups for COVID-19 hospitalization and severe COVID-19.

## 1. Introduction

The coronavirus disease 2019 (COVID-19) caused by a new type of coronavirus, namely Severe Acute Respiratory Syndrome Coronavirus 2 (SARS-CoV-2), has been declared a global pandemic [1]. Despite that COVID-19 is a mild condition in most individuals, it can be life-threatening for others [2]. Several studies have reported the increased odds of having chronic comorbidities in patients with severe COVID-19 [3,4,5]. Hypertension and hypertension-related major chronic conditions, such as cardiovascular diseases (CVD), cerebrovascular diseases (CBVD) and chronic kidney disease (CKD) [6], were described among prevalent underlying conditions in COVID-19 patients [3,5,7].

The independent role of hypertension and hypertension-related conditions on COVID-19 hospitalization and severe COVID-19 remains unclear. Univariate analyses have shown an association with severe COVID-19 [3,8,9]. However, as the prevalence of hypertension and other comorbidities increases with age [6,10], and the burden of COVID-19 has been especially remarkable in older ages [8,11] and among those with major chronic conditions [12,13], the association of hypertension and related conditions with severe COVID-19 may be due to older age or other chronic conditions. Only few investigations have adjusted for potential confounders, and have not found hypertension as an independent risk factor for severe disease among COVID-19 hospitalized patients [14,15], whereas hypertension-related conditions, such as CVD [15,16] and CKD [14,16,17], have been found as risk factors. Only one study has evaluated the effect of hypertension on the risk of COVID-19 mortality in the population, suggesting that hypertension could be a protective factor, while chronic heart disease and reduced kidney function increased the risk [18]. However, no study has addressed the effect of hypertension on the risk of COVID-19 hospitalization and severe outcomes in the general population.

The overwhelming situation in hospitals due to COVID-19 pandemic, the high proportion of individuals with these conditions in society [6,19,20], and the lack of scientific evidence make such an evaluation timely. Therefore, in a population-based cohort, the current study aimed to evaluate hypertension, CVD, CBVD, and CKD as independent risk factors for laboratory-confirmed COVID-19 hospitalization and for severe COVID-19, defined as intensive care unit (ICU) admission or death. Additionally, the risk of severe disease among COVID-19 hospitalized patients was also evaluated.

## 2. Materials and Methods

### 2.1. Study Design and Setting

**Figure 1:**
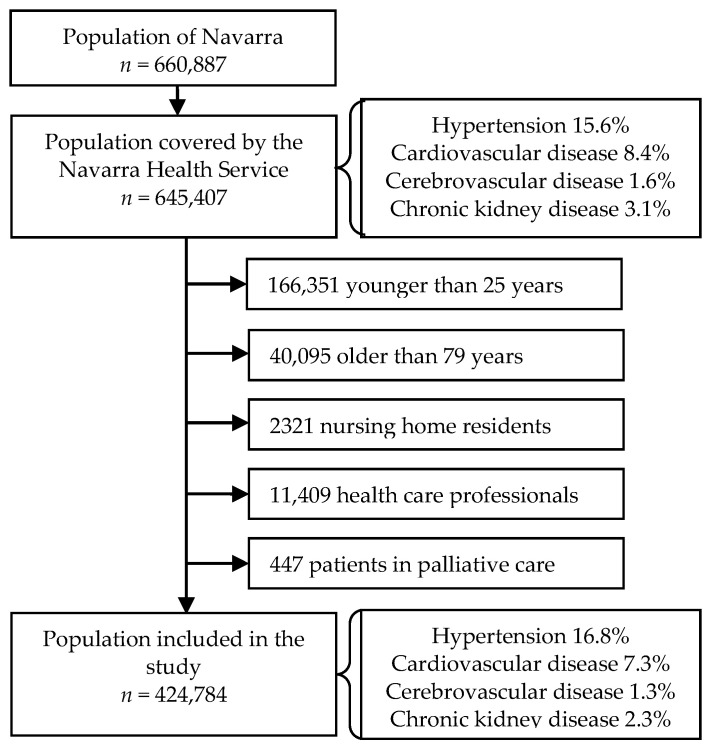
Flow chart of the cohort definition.

A prospective population-based cohort study was performed in Navarra, a Spanish region of ~660,000 inhabitants. Navarra Health Service provides health care, free at the point of service, to 97% of the population of the region. Improvements in the treatment of COVID-19 patients were introduced according to the current scientific recommendations and guidelines. By the end of February 2020, when the first case of COVID-19 was confirmed in the region, we defined the cohort of the population aged 25 to 79 years and covered by the Health Service. Healthcare professionals, nursing home residents, and patients in palliative care were excluded from the current analysis, leaving a total of 424,784 individuals (Figure 1). The Ethical Committee for Clinical Research of Navarra approved the study protocol (approval code: PI2020/45; date: 8 May 2020).

### 2.2. Data Collection

Sociodemographic characteristics, health care use, smoking status, and the diagnosis of hypertension and major chronic conditions referred to at the beginning of the follow-up (1 March 2020) were obtained from the electronic medical records. This source of information has demonstrated high sensitivity and specificity to detect chronic medical conditions [21].

Individuals with hypertension were considered to be all prevalent cases that had had this diagnosis established by a general practitioner and had been coded as K86 and K87 in the International Classification of Primary Care version 2 [22]. CVD included those diseases coded as K71, K74–K77, K81–K84, and K99; CBVD those classified as K90 and K91; and CKD those coded as U99. Supplementary Table S1 shows conditions included in each of the codes. Since CVD, CBVD, and CKD frequently concur with hypertension, to simplify the wording, they were labeled as “hypertension-related conditions” in the current study, regardless of whether hypertension was present or was the cause of these conditions.

Sociodemographic variables included gender, age, origin country (Spain or other), municipality population (<5000, 5000–100,000, and >100,000 inhabitants), and annual taxable income level (none or dependent, <18,000, 18,000–100,000 and >100,000 €).

Health care use included the number of visits to primary healthcare centers and hospitalizations in the prior 12 months. Smoking status was categorized as non-smoker, former smoker, current smoker, and unknown. Other major chronic conditions considered were diabetes, chronic obstructive pulmonary disease, asthma, liver cirrhosis, immunodeficiency, and morbid obesity (body mass index ≥ 40 kg/m^2^). The lack of diagnosis of hypertension or a chronic disease was considered as not having that condition.

### 2.3. Outcomes

Since the beginning of March 2020, all hospitalized patients with clinical manifestation compatible with COVID-19 [23] were tested for SARS-CoV-2 using commercial kits based on reverse transcription quantitative real-time polymerase-chain-reaction (RT-qPCR) in an upper respiratory tract sample and some were also tested by antibody rapid test (Wondfo, Guangzhou, China) in a blood sample. Patients tested positive for RT-qPCR or for antibody rapid test from March to May 2020, who were hospitalized or died of COVID-19, were considered as events of interest for this study. COVID-19 hospitalized cases included those admitted for 24 h or more and those who died in the emergency room before admission. Confirmed patients who were admitted to ICU or who died of COVID-19 in the hospital or within 30 days after hospital admission were considered COVID-19 severe cases. Hospital and ICU admissions were obtained from the Health Service electronic records. Deaths were obtained from electronic medical records and administrative databases and were validated with the Daily Mortality Monitoring System. As a part of the epidemiological surveillance in the region, hospital admissions and deaths had been reviewed by public health doctors to detect those related to COVID-19, and only these hospitalizations and deaths were considered for the present study. The database was anonymized before the analysis.

### 2.4. Statistical Analysis

Characteristics of individuals with either hypertension, CVD, CBVD, or CKD were compared to characteristics of those persons in the cohort free of the assessed condition by the chi-square test.

The incidence of COVID-19 hospitalization and severe COVID-19 (ICU admission and exitus, both separately and jointly) per 100,000 inhabitants were calculated by the presence of hypertension and hypertension-related conditions. In the whole study population, Poisson regression was used to assess the independent effect of each condition for all analyzed COVID-19 outcomes. Among patients hospitalized for COVID-19, Poisson regression was used to evaluate the independent effects of hypertension and each hypertension-related condition as predictors of severe outcomes. Crude and adjusted relative risks (RR) with their 95% confidence intervals (CI) were calculated. A partially-adjusted analysis considered socio-demographic variables only, and the fully-adjusted analysis also included health care use, smoking status, hypertension, the three hypertension-related conditions, and other major chronic conditions. The independent effect of hypertension was also explored in the analysis stratified by the diagnosis of any hypertension-related condition. Interaction terms of sex and age with hypertension and related conditions were tested and ruled out.

Analyses were performed using STATA/SE V.15 (StataCorp, College Station, Texas, USA). *p* values < 0.05 were considered as statistically significant.

## 3. Results

### 3.1. Characteristics of the Study Population

The study population included 424,784 persons aged 25 to 79 years; 71,323 (16.8%) of them had been previously diagnosed with hypertension, 31,005 (7.3%) with CVD, 5648 (1.3%) with CBVD, and 9901 (2.3%) with CKD (Figure 1). Compared to the rest of the population, individuals with hypertension were more frequently men (55.9% vs 49.5%; *p* < 0.001), aged 65 years or more (55.7% vs 13.0%; *p* < 0.001), born in Spain (90.3% vs 79.6%; *p* < 0.001), had visited a primary healthcare center five or more times (57.1% vs 34.6%; *p* < 0.001), and had been hospitalized (9.0% vs 4.8%; *p* < 0.001) during the previous year. Individuals with hypertension had a higher prevalence of CVD (16.9% vs 5.4%; *p* < 0.001), CBVD (4.3% vs 0.7%; *p* < 0.001), and CKD (8.1% vs 1.2%; *p* < 0.001), as well as other major chronic condition (39.3% vs 16.0%; *p* < 0.001). Similar differences were also observed when comparing characteristics of persons with each of the hypertension-related conditions to those free of the respective disease (all comparisons, *p* < 0.05). Hypertension was present in 38.9% of people with CVD, 53.9% of those with CBVD, and 58.2% of those with CKD (Table 1).

### 3.2. COVID-19 Hospitalization and Severe Outcomes

A total of 1106 individuals were hospitalized for confirmed COVID-19 (260 per 100,000 population) and 176 were severe cases (41 per 100,000). Of these, 117 were admitted to ICU and 97 died (38 died in ICU) (Table 2). The mean age was 50.5, 60.2, 63.3, and 71.6 years for the study population, hospitalized cases, ICU admitted patients and those who died, respectively. While subjects with hypertension were 16.8% of the study population, they resulted in 32.0% of the COVID-19 hospitalizations, 44.4% of the ICU admissions, and 56.7% of the COVID-19 deaths. Similarly, those with hypertension-related conditions were highly represented among the COVID-19 hospitalized individuals and severe COVID-19 cases (Supplementary Table S2).

As compared to persons without hypertension, the crude RR of those with hypertension was 2.33 (95% CI 2.06–2.65, *p* < 0.001) for COVID-19 hospitalization and 4.63 (95% CI 3.44–6.22, *p* < 0.001) for severe COVID-19; specifically, the crude RR was 3.96 (95% CI 2.75–5.71, *p* < 0.001) for ICU admission and 6.49 (95% CI 4.34–9.70, *p* < 0.001) for COVID-19 mortality. The crude risks for all evaluated outcomes were significantly higher among those with each of the assessed hypertension-related conditions as compared to persons free of that condition (Table 2).

### 3.3. Hypertension, Related Conditions, and Risk for COVID-19 Outcomes

When adjusting for socio-demographic characteristics only, the RRs of hypertension for COVID-19 hospitalization and severe COVID-19 declined but remained statistically significant (partially-adjusted RR 1.22, 95% CI 1.06–1.41, *p* = 0.005; RR 1.53, 95% CI 1.11–2.10, *p* = 0.009; respectively). Nevertheless, further adjustment for health-related baseline conditions made the effect of hypertension not significant with fully-adjusted RR (aRR) 0.96 (95% CI 0.83–1.12, *p* = 0.622), aRR 1.38 (95% CI 0.91–2.09, *p* = 0.124) and aRR 1.09 (95% CI 0.70–1.67, *p* = 0.734) for COVID-19 hospitalization, ICU admission and exitus, respectively (Table 3).

In the fully-adjusted analyses, CVD, CBVD and CKD were statistically associated with higher risk of COVID-19 hospitalization (aRR 1.33, 95% CI 1.13–1.58, *p* < 0.001; aRR 1.41, 95% CI 1.04–1.92, *p* = 0.025 and aRR 1.52, 95% CI 1.21–1.91, *p* < 0.001; respectively), of severe COVID-19 (aRR 1.61, 95% CI 1.13–2.30, *p* = 0.008; aRR 1.91, 95% CI 1.13–3.25, *p* = 0.016; and aRR 1.78, 95% CI 1.14–2.76, *p* = 0.010; respectively) and specifically of mortality (aRR 2.17, 95% CI 1.40–3.37, *p* = 0.001; aRR 2.76, 95% CI 1.58–4.81, *p* < 0.001; and aRR 1.83, 95% CI 1.09–3.07, *p* = 0.022; respectively). Point estimates indicated the three conditions as independent predictors of ICU admission, but the association was not statistically significant (Table 3).

The possible effect of hypertension on COVID-19 outcomes was evaluated in a further analysis, stratifying by the diagnosis of any of the three hypertension-related conditions. The fully-adjusted model showed no association between hypertension and risk of COVID-19 hospitalization in those without (aRR 0.95, 95% CI 0.80–1.14, *p* = 0.589) and with hypertension-related chronic conditions (aRR 0.99, 95% CI 0.76–1.30, *p* = 0.958). No statistically significant association was detected between hypertension and severe COVID-19 in persons without and with the diagnosis of hypertension-related conditions (Table 4).

### 3.4. Effect on Severe Outcomes among COVID-19 Hospitalized Patients

Among patients hospitalized for COVID-19, hypertension and each hypertension-related condition were associated with an increased risk for COVID-19 exitus in the crude analyses, but only CVD and CBVD had a statistically significant effect in the partially-adjusted analysis (aRR 1.84, 95% CI 1.21–2.81, *p* = 0.005; and aRR 2.23, 95% CI 1.27–3.91, *p* = 0.005; respectively). Only CBVD maintained a statistically significant effect in the fully-adjusted analysis (aRR 1.80, 95% CI 1.00–3.23, *p* = 0.049). No statistically significant effect on ICU admission was observed (Table 5).

## 4. Discussion

To the best of our knowledge, this is the first study evaluating the independent effect of hypertension and related conditions for COVID-19 hospitalization and severity in the general population. In a prospective population-based cohort, the present study shows that CVD, CBVD and CKD, but not hypertension per se, were independent predictors for both COVID-19 hospitalization and COVID-19 mortality. These results have very important practical implications, not only for hospital clinicians but also for general practitioners and public health professionals, due to the high prevalence of these conditions in the population [6,19,20] and among COVID-19 patients, as the current and previous studies have pointed out [3,5,24].

The only previous population-based cohort study evaluating the effect of hypertension on severe COVID-19 analyzed mortality only, and curiously found hypertension as a preventive factor for this outcome without a clear explanation. In the same study, chronic heart diseases and reduced kidney function were independent risk factors for COVID-19 mortality [18].

We found that hypertension is a risk factor for COVID-19 hospitalization and severe disease in the general population in the crude analysis and in the partially-adjusted analysis that only included age, sex and other sociodemographic variables. However, this association lost statistical significance when major chronic conditions and health care service use were considered in the model. A different effect of hypertension on COVID-19 mortality was also described in the partially- and fully-adjusted analyses of the English cohort study [18]. This seems to indicate that hypertension is indirectly associated with a higher risk for COVID-19 hospitalization and severity in the population, and that this risk excess could be mediated by the presence of major chronic conditions.

When specifically assessing hypertension-related conditions, i.e., CVD, CBVD, and CKD, we observed that all of them were independent risk factors for hospitalization, severe disease, and exitus by COVID-19 in the population. These findings complement results of previous studies that found CKD as an independent risk factor for hospitalization [16,25] and mortality [25] among COVID-19-confirmed individuals, while contradictory findings have been reported for the role of CVD [16,25]. The effect of chronic heart disease and reduced kidney function as risk factors for COVID-19 related mortality in the population has been previously suggested [18].

The current study confirms the findings of those previous investigations assessing COVID-19 hospitalized patients. The crude analysis indicated that COVID-19 hospitalized individuals with hypertension were at significantly higher risk of severe COVID-19, both ICU admission and mortality [26,27], but further adjustment made the association not significant [13,14,15]. Another study has even suggested that hypertension could be a protective factor for the composite outcome of venous thromboembolism or mortality [28]. Although point estimates suggest CVD, CBVD and CKD as possible risk factors for COVID-19 mortality, in the fully-adjusted analysis the association was only statistically significant for CBVD. Some previous studies have suggested CVD and CKD as risk factors for COVID-19 mortality [13,15,28], but others have not [14,16,28]. The effect of CBVD has been recently reported [28].

Besides viral pneumonia, severe COVID-19 cases may also present multi-organ damage, showing cardiac complications [24,29,30,31], thromboembolism and vascular disorders [29,30,32], and acute kidney injury [17,24,33]. Thus, it seems reasonable that those individuals with previous related pathologies could be at higher risk of mortality.

We did not detect a significant association of hypertension, CVD, CBVD, and CKD with ICU admission due to COVID-19 in the population and in COVID-19 hospitalized patients. Although point estimates may suggest small effects, statistical power was low in this analysis. Patients with hypertension or related chronic conditions did not specifically develop acute respiratory failure, which is the main cause of ICU admission in COVID-19 patients [34]. The presence of severe chronic conditions and older age in COVID-19 patients may also discourage ICU admission.

The main strengths of our study are the prospective cohort design, the population representativeness, the relatively large size of the studied population, and that only laboratory-confirmed cases were considered. Additionally, cases only included patients in whom COVID-19 was the main reason for hospitalization or were experiencing a severe outcome; nevertheless, it is possible that some deaths due to COVID-19 could have been missed if they occurred outside the hospital. Analyses were adjusted for most potentially confounding variables. Information about the presence of the analyzed diseases was obtained from clinical records before the beginning of the follow-up to prevent information bias.

Some limitations should also be mentioned. The hospitalization and severe COVID-19 risk assessment includes the combined risk of both infection and the experience of complications relevant enough to require hospitalization. Future studies considering the prevalence of SARS-CoV-2 specific antibodies could help to determine the specific effect of the assessed comorbidities on the infection risk. Persons aged under 25 and over 79 years, healthcare professionals, nursing home residents, and patients in palliative care were excluded from the analysis to improve the internal validity and the presentation of results, since they may have a different epidemiological pattern and use of healthcare services; therefore, the results and conclusions are not applicable to these groups. The current study compares persons with a diagnosis of the assessed conditions with those without that diagnosis. As it is possible that some individuals were not diagnosed but actually had the condition, our findings could be seen as an underestimation of the effect. The severity of comorbidities, treatments for hypertension and comorbidities, and the treatment received at the hospital were not available.

## 5. Conclusions

The results of this prospective population-based cohort study show that hypertension per se is not a risk factor for COVID-19, but those individuals with major chronic conditions frequently associated with hypertension, i.e., CVD, CBVD and CKD, should be recognized at increased risk for both hospitalization and death from COVID-19. The presence of CBVD would also lead to an increased risk of mortality among COVID-19 hospitalized patients. Since hypertension may be the cause of these major chronic conditions, and through them, indirectly associated with COVID-19 hospitalization and mortality, policies to tackle the hypertension pandemic could have benefits for fighting both non-communicable and infectious diseases.

## Figures and Tables

**Table 1:** Characteristics (in percentages) of the cohort population with and without prior diagnosis of hypertension, cardiovascular disease, cerebrovascular disease and chronic kidney disease.

Socio-Demographic and Clinical Variables	Hypertension		Cardiovascular Disease		Cerebrovascular Disease		Chronic Kidney Disease	
	No	Yes	No	Yes	No	Yes	No	Yes
Population, number	353,461	71,323	393,779	31,005	419,136	5648	414,883	9901
Sex								
Female	50.5	44.1	50.3	37.5	49.6	36.8	49.6	38.7
Male	49.5	55.9	49.7	62.5	50.4	63.2	50.4	61.3
Age, years								
25–49	57.7	9.2	51.7	22.6	50.1	8.2	50.5	10.9
50–64	29.3	35.1	30.3	29.0	30.3	29.3	30.4	22.4
65–79	13.0	55.7	18.0	48.3	19.6	62.4	19.1	66.7
Country of origin								
Spain	79.6	90.3	80.7	90.9	81.3	92.0	81.2	92.7
Other	20.4	9.7	19.3	9.1	18.7	8.0	18.8	7.3
Municipality population								
>100,000	31.8	32.4	31.8	33.6	31.9	33.6	31.8	34.1
5000–100,000	34.8	32.4	34.5	33.3	34.5	31.6	34.5	31.4
<5000	33.4	35.1	33.7	33.1	33.7	34.9	33.7	34.6
Annual taxable income level (€)								
None/dependent	5.9	5.4	5.9	5.2	5.9	5.7	5.9	5.6
<18,000	53.4	51.0	53.3	49.0	52.9	54.7	53.0	52.3
18,000–100,000	40.0	42.9	40.2	45.1	40.5	39.0	40.5	41.6
>100,000	0.7	0.7	0.7	0.7	0.7	0.7	0.7	0.4
Primary healthcare visits in prior 12 months								
0	22.4	8.0	20.9	8.6	20.2	5.0	20.3	5.5
1–4	43.0	34.9	42.2	34.2	41.8	29.5	41.9	28.9
5–9	19.9	27.3	20.7	26.1	21.0	27.2	21.0	27.9
>9	14.7	29.8	16.2	31.0	17.0	38.3	16.8	37.7
Hospitalization in prior 12 months	4.8	9.0	4.9	12.7	5.3	18.3	5.3	14.1
Smoking status								
Current smoker	11.7	29.0	13.9	23.3	14.4	25.7	14.3	28.5
Former smoker	3.7	11.4	4.5	11.6	4.9	13.4	4.8	12.1
Never smoker	17.5	20.6	17.9	20.4	18.0	22.9	18.0	18.6
Unknown	67.1	39.1	63.8	44.7	62.7	37.9	62.9	40.8
Hypertension	0	100.0	15.0	38.9	16.3	53.9	15.8	58.2
Hypertension-related conditions								
Cardiovascular disease	5.4	16.9	0	100.0	7.1	25.3	6.8	26.9
Cerebrovascular disease	0.7	4.3	1.1	4.6	0	100.0	1.2	6.3
Chronic kidney disease	1.2	8.1	1.8	8.6	2.2	11.0	0	100.0
Any other major chronic condition	16.0	39.3	18.6	36.6	19.6	42.6	19.3	46.7

**Table 2:** Cases and incidence rates ^1^ of confirmed COVID-19 that were admitted to the hospital, to intensive care unit or who died, according to the prior diagnosis of hypertension, cardiovascular disease, cerebrovascular disease or chronic kidney disease.

		Hospitalization	Severe Cases ^2^	ICU Admission	Deaths
Study population	N	1106	176	117	97
	Rate ^1^	260	41	28	23
Persons with HT	N	354	85	52	55
	Rate ^1^	496	119	73	77
Persons without HT	N	752	91	65	42
	Rate ^1^	213	26	18	12
HT vs all others	RR (95% CI)	2.33 (2.06–2.65)	4.63 (3.44–6.22)	3.96 (2.75–5.71)	6.49 (4.34–9.70)
Persons with CVD	N	188	51	25	41
	Rate ^1^	606	164	81	132
Persons without CVD	N	918	125	92	56
	Rate ^1^	233	32	23	14
CVD vs all others	RR (95% CI)	2.60 (2.22–3.04)	5.18 (3.74–7.18)	3.45 (2.22–5.37)	9.30 (6.22–13.91)
Persons with CBVD	N	46	16	5	16
	Rate ^1^	814	283	89	283
Persons without CBVD	N	1060	160	112	81
	Rate ^1^	253	38	27	19
CBVD vs all others	RR (95% CI)	3.22 (2.40–4.33)	7.42 (4.44–12.41)	3.31 (1.35–8.11)	14.66 (8.57–25.06)
Persons with CKD	N	89	27	10	21
	Rate ^1^	899	273	101	212
Persons without CKD	N	1017	149	107	76
	Rate ^1^	245	36	26	18
CKD vs all others	RR (95% CI)	3.67 (2.95–4.55)	7.59 (5.04–11.44)	3.92 (2.05–7.49)	11.58 (7.14–18.77)

^1^ Rates are expressed as cases per 100,000 persons. ^2^ Severe cases include patients who were admitted to intensive care unit or died. ICU, intensive care unit; RR, relative risk; CI, confidence interval; HT, hypertension; CVD, cardiovascular disease; CBVD, cerebrovascular disease; CKD, chronic kidney disease.

**Table 3:** Association of hypertension and hypertension-related conditions (cardiovascular, cerebrovascular or chronic kidney diseases) with COVID-19 outcomes.

	Crude Analysis		Partially Adjusted Analysis ^1^		Fully Adjusted Analysis ^2^	
	RR (95% CI)	*p-*Value	RR (95% CI)	*p-*Value	RR (95% CI)	*p-*Value
Analysis of hospitalizations						
Hypertension	2.33 (2.06–2.65)	<0.001	1.22 (1.06–1.41)	0.005	0.96 (0.83–1.12)	0.622
Cardiovascular disease	2.60 (2.22–3.04)	<0.001	1.65 (1.40–1.94)	<0.001	1.33 (1.13–1.58)	<0.001
Cerebrovascular disease	3.22 (2.40–4.33)	<0.001	1.74 (1.29–2.35)	<0.001	1.41 (1.04–1.92)	0.025
Chronic kidney disease	3.67 (2.95–4.55)	<0.001	1.97 (1.57–2.46)	<0.001	1.52 (1.21–1.91)	<0.001
Analysis of severe cases ^3^						
Hypertension	4.63 (3.44–6.22)	<0.001	1.53 (1.11–2.10)	0.009	1.12 (0.80–1.56)	0.517
Cardiovascular disease	5.18 (3.74–7.18)	<0.001	2.22 (1.58–3.12)	<0.001	1.61 (1.13–2.30)	0.008
Cerebrovascular disease	7.42 (4.44–12.41)	<0.001	2.60 (1.54–4.40)	<0.001	1.91 (1.13–3.25)	0.016
Chronic kidney disease	7.59 (5.04–11.44)	<0.001	2.60 (1.70–3.99)	<0.001	1.78 (1.14–2.76)	0.010
Analysis of ICU admission						
Hypertension	3.96 (2.75–5.71)	<0.001	1.75 (1.18–2.59)	0.005	1.38 (0.91–2.09)	0.124
Cardiovascular disease	3.45 (2.22–5.37)	<0.001	1.84 (1.66–2.91)	0.009	1.44 (0.90–2.31)	0.131
Cerebrovascular disease	3.31 (1.35–8.11)	0.009	1.55 (0.63–3.84)	0.341	1.18 (0.47–2.93)	0.729
Chronic kidney disease	3.92 (2.05–7.49)	<0.001	1.88 (0.97–3.64)	0.064	1.28 (0.65–2.53)	0.476
Analysis of exitus						
Hypertension	6.49 (4.34–9.70)	<0.001	1.53 (1.01–2.32)	0.047	1.09 (0.70–1.67)	0.734
Cardiovascular disease	9.30 (6.22–13.91)	<0.001	3.16 (2.08–4.79)	<0.001	2.17 (1.40–3.37)	0.001
Cerebrovascular disease	14.66 (8.57–25.06)	<0.001	3.89 (2.25–6.73)	<0.001	2.76 (1.58–4.81)	<0.001
Chronic kidney disease	11.58 (8.57–18.77)	<0.001	2.87 (1.74–4.72)	<0.001	1.83 (1.09–3.07)	0.022

^1^ Adjusted for sociodemographic characteristics: sex, age, country of origin, municipality population and annual taxable income level. ^2^ Additionally adjusted for health-related characteristics: hypertension, cardiovascular disease, cerebrovascular disease, chronic kidney disease, primary health care visits in prior 12 months, hospitalization in prior 12 months, smoking status, and other major chronic conditions. ^3^ Severe cases include patients who were admitted to intensive care unit or died. RR, relative risk; CI, confidence interval; ICU, intensive care unit.

**Table 4:** Effect of hypertension on the risk of hospitalization, severe outcomes ^1^, admission to intensive care unit (ICU) or death for COVID-19 by the presence of any hypertension-related conditions (cardiovascular, cerebrovascular or chronic kidney diseases).

COVID-19 Outcome Evaluated	Crude Analysis		Partially-Adjusted Analysis ^2^		Fully-Adjusted Analysis ^3^	
	RR (95% CI)	*p-*Value	RR (95% CI)	*p-*Value	RR (95% CI)	*p-*Value
People without hypertension-related conditions						
Hospitalization	2.01 (1.72–2.35)	<0.001	1.13 (0.95–1.34)	0.167	0.95 (0.80–1.14)	0.589
Severe cases ^1^	3.61 (2.45–5.30)	<0.001	1.32 (0.87–1.98)	0.189	1.10 (0.72–1.69)	0.658
ICU admission	3.76 (2.44–5.80)	<0.001	1.68 (1.06–2.67)	0.027	1.38 (0.85–2.23)	0.192
Exitus	5.17 (2.90–9.24)	<0.001	1.33 (0.72–2.43)	0.363	1.11 (0.59–2.07)	0.751
People with any hypertension-related condition						
Hospitalization	1.74 (1.36–2.23)	<0.001	1.20 (0.93–1.56)	0.161	0.99 (0.76–1.30)	0.958
Severe cases ^1^	2.84 (1.69–4.78)	<0.001	1.58 (0.92–2.70)	0.097	1.21 (0.70–2.10)	0.499
ICU admission	2.35 (1.12–4.93)	0.025	1.72 (0.79–3.75)	0.174	1.46 (0.65–3.26)	0.361
Exitus	2.72 (1.52–4.86)	0.001	1.31 (0.73–2.38)	0.365	1.05 (0.57–1.92)	0.885

^1^ Severe cases include patients who were admitted to intensive care unit or died. ^2^ Adjusted for sociodemographic characteristics: sex, age, country of origin, municipality population and annual taxable income level. ^3^ Additionally adjusted for health-related characteristics: cardiovascular disease, cerebrovascular disease, chronic kidney disease, primary health care visits in prior 12 months, hospitalization in prior 12 months, smoking status, and other major chronic conditions. RR, relative risk; CI, confidence interval; ICU, intensive care unit.

**Table 5:** Effect of hypertension and hypertension-related conditions on severe outcomes ^1^ among patients hospitalized for COVID-19.

Chronic Condition Evaluated	Crude Analysis		Partial-Adjusted Analysis ^2^		Fully-Adjusted Analysis ^3^	
	RR (95% CI)	*p-*Value	RR (95% CI)	*p-*Value	RR (95% CI)	*p-*Value
Severe cases						
Hypertension	1.98 (1.48–2.67)	<0.001	1.31 (0.96–1.79)	0.092	1.19 (0.86–1.65)	0.298
Cardiovascular disease	1.99 (1.44–2.76)	<0.001	1.20 (0.84–1.72)	0.321	1.14 (0.79–1.65)	0.474
Cerebrovascular disease	2.30 (1.38–3.85)	0.002	1.34 (0.78–2.30)	0.289	1.26 (0.73–2.18)	0.407
Chronic renal disease	2.07 (1.37–3.12)	<0.001	1.21 (0.78–1.87)	0.392	1.13 (0.73–1.76)	0.581
ICU admission						
Hypertension	1.70 (1.18–2.45)	0.004	1.42 (0.96–2.10)	0.077	1.42 (0.94–2.13)	0.095
Cardiovascular disease	1.33 (0.85–2.06)	0.210	1.07 (0.66–1.72)	0.790	1.05 (0.64–1.70)	0.856
Cerebrovascular disease	1.03 (0.42–2.52)	0.951	0.91 (0.36–2.30)	0.850	0.87 (0.34–2.23)	0.778
Chronic renal disease	1.07 (0.56–2.04)	0.842	0.86 (0.44–1.69)	0.662	0.82 (0.41–1.62)	0.565
Exitus						
Hypertension	2.78 (1.86–4.16)	<0.001	1.38 (0.91–2.09)	0.131	1.15 (0.75–1.79)	0.518
Cardiovascular disease	3.58 (2.39–5.35)	<0.001	1.84 (1.21–2.81)	0.005	1.45 (0.92–2.30)	0.111
Cerebrovascular disease	4.55 (2.66–7.78)	<0.001	2.23 (1.27–3.91)	0.005	1.80 (1.00–3.23)	0.049
Chronic renal disease	3.16 (1.95–5.12)	<0.001	1.51 (0.92–2.50)	0.105	1.10 (0.65–1.86)	0.714

^1^ Severe outcomes include patients who were admitted to intensive care unit or died. ^2^ Adjusted for sociodemographic characteristics: sex, age, country of origin, municipality population and annual taxable income level. ^3^ Additionally adjusted for health-related characteristics: cardiovascular disease, cerebrovascular disease, chronic kidney disease, primary health care visits in prior 12 months, hospitalization in prior 12 months, smoking status, and other major chronic conditions. RR, relative risk; CI, confidence interval; ICU, intensive care unit.

## Supplementary Materials

The following are available online at https://www.mdpi.com/2077-0383/10/6/1194/s1, Table S1: Codes of the International Classification of Primary Care version 2 considered in the current study, Table S2: Characteristics of the study population, and patients with the assessed COVID-19 outcomes (hospitalization, severe case, intensive care unit admission and death).
