# Supplementary material for: Hypertension and Related Comorbidities as Potential Risk Factors for COVID-19 Hospitalization and Severity: A Prospective Population-Based Cohort Study
Source: J Clin Med. 2021 Mar 12;10(6):1194. doi: 10.3390/jcm10061194 (PMC8000595; doi:10.3390/jcm10061194)
Supplement: Supplementary file 1 [file jcm-10-01194-s001.pdf]

Supplementary Material

Supplementary Table S1. Codes of the International Classification of Primary Care version 2 [1] considered in the current study.

Condition	Code	Description
Hypertension	K86	Hypertension uncomplicated
	K87	Hypertension complicated
Cardiovascular disease	K71	Rheumatic fever/heart disease
	K74	Ischaemic heart disease with angina
	K75	Acute myocardial infarction
	K76	Ischaemic heart disease without angina
	K77	Heart failure
	K81	Heart/arterial murmur not otherwise specified
	K82	Pulmonary heart disease
	K83	Heart valve disease not otherwise specified
	K84	Heart disease other
	K99	Cardiovascular disease other
Cerebrovascular disease	K90	Stroke/cerebrovascular accident
	K91	Cerebrovascular disease
Chronic kidney disease	U99	Urinary disease, other

Supplementary Table S2. Characteristics of the study population, and patients with the assessed COVID-19 outcomes (hospitalization, severe case, intensive care unit admission and death).

Characteristics	Population<br>n = 424,784<br>%	Hospitalized patients<br>n =1,106<br>%	Severe cases<br>n = 176<br>%	Intensive care unit admitted<br>n = 117<br>%	Deaths<br>n = 97<br>%
**Sex**					
Female	49.4	43.1	29.5	29.9	28.9
Male	50.6	56.9	70.5	70.1	71.1
**Age, years**					
25-49	49.6	23.6	6.3	9.4	1.0
50-64	30.3	32.7	28.4	38.5	15.5
65-79	20.2	43.7	65.3	52.1	83.5
**Country of origin**					
Spain	81.5	79.7	90.3	86.3	93.8
Other	18.6	20.3	9.7	13.7	6.2
**Municipality population**					
>100,000	31.9	38.4	33.5	34.2	32.0
5000-100,000	34.4	34.7	37.5	39.3	33.0
<5000	33.7	26.9	29.0	26.5	35.1
**Annual taxable income level (€)**					
None/dependent	5.9	6.5	7.4	10.3	6.2
<18,000	53.0	48.7	46.6	36.8	59.8
18,000-100,000	40.5	42.9	43.2	49.6	33.0
>100,000	0.7	1.9	2.8	3.4	1.0
**Primary healthcare visits in prior 12 months**					
0	20.0	9.8	9.1	9.4	7.2
1-4	41.7	32.9	27.8	34.2	23.7
5-9	21.1	26.9	27.3	27.4	26.8
>9	17.3	30.4	35.8	29.1	42.3
**Hospitalization in prior 12 months**	5.5	9.6	15.3	10.3	22.7
**Smoking status**					
Current smoker	14.6	24.5	22.7	22.2	26.8
Former smoker	5.0	9.5	12.5	8.5	15.5
Never smoker	18.1	10.5	19.3	18.8	19.6
Unknown	62.4	55.5	45.5	50.4	38.1
**Hypertension**	16.8	32.0	48.3	44.4	56.7
**Hypertension-related conditions**					
Cardiovascular disease	7.3	17.0	29.0	21.4	42.3
Cerebrovascular disease	1.3	4.2	9.1	4.3	16.5
Chronic kidney disease	2.3	8.0	15.3	8.5	21.6
**Any other major chronic condition**	19.9	36.3	54.0	49.6	62.9

Reference

1. International Classification Committee of WONCA. International Classification of Primary Care – Revised 2nd Edition. Oxford University Press: Oxford, **2005**.
